# Automatic measurements of fetal intracranial volume from 3D ultrasound scans

**DOI:** 10.3389/fnimg.2022.996702

**Published:** 2022-11-04

**Authors:** Yaron Caspi, Sonja M. C. de Zwarte, Iris J. Iemenschot, Raquel Lumbreras, Roel de Heus, Mireille N. Bekker, Hilleke Hulshoff Pol

**Affiliations:** ^1^Department of Psychiatry, UMC Utrecht Brain Center, University Medical Center Utrecht, Utrecht, Netherlands; ^2^Department of Obstetrics and Gynaecology, St. Antonius Hospital, Utrecht, Netherlands; ^3^Department of Obstetrics, University Medical Center Utrecht, Utrecht, Netherlands; ^4^Department of Psychology, Utrecht University, Utrecht, Netherlands

**Keywords:** ultrasound, intracranial volume (ICV), automatic analysis, fetal brain development, registration

## Abstract

Three-dimensional fetal ultrasound is commonly used to study the volumetric development of brain structures. To date, only a limited number of automatic procedures for delineating the intracranial volume exist. Hence, intracranial volume measurements from three-dimensional ultrasound images are predominantly performed manually. Here, we present and validate an automated tool to extract the intracranial volume from three-dimensional fetal ultrasound scans. The procedure is based on the registration of a brain model to a subject brain. The intracranial volume of the subject is measured by applying the inverse of the final transformation to an intracranial mask of the brain model. The automatic measurements showed a high correlation with manual delineation of the same subjects at two gestational ages, namely, around 20 and 30 weeks (linear fitting R^2^(20 weeks) = 0.88, R^2^(30 weeks) = 0.77; Intraclass Correlation Coefficients: 20 weeks=0.94, 30 weeks = 0.84). Overall, the automatic intracranial volumes were larger than the manually delineated ones (84 ± 16 vs. 76 ± 15 cm^3^; and 274 ± 35 vs. 237 ± 28 cm^3^), probably due to differences in cerebellum delineation. Notably, the automated measurements reproduced both the non-linear pattern of fetal brain growth and the increased inter-subject variability for older fetuses. By contrast, there was some disagreement between the manual and automatic delineation concerning the size of sexual dimorphism differences. The method presented here provides a relatively efficient way to delineate volumes of fetal brain structures like the intracranial volume automatically. It can be used as a research tool to investigate these structures in large cohorts, which will ultimately aid in understanding fetal structural human brain development.

## 1. Introduction

Fetal brain development is critical for the child's functioning during the neonatal period and later in life (Stiles and Jernigan, 2010). It is characterized by complex molecular and cellular processes, and a disruption of these processes can have severe consequences (Raybaud et al., 2013; Teli et al., 2018). Both genetic and environmental factors that interfere with normal fetal brain growth are associated with severe developmental disorders (Sun and Hevner, 2014; Li et al., 2018). Moreover, some adulthood manifested conditions are suspected of having a fetal origin, especially among subjects in a low percentile of birth weight (Lærum et al., 2017). Consequently, the theory of Development and Origins of Health and Disease (DOHaD) suggests that processes during fetal growth can cause physical and mental adversaries in childhood, adolescence, and even adulthood (Heindel and Vandenberg, 2015).

Whether the relationship between fetal development and functioning in later life holds over the whole range of fetal growth values or is restricted to extreme growth abnormalities is still not fully settled (Schlotz and Phillips, 2009). Some evidence suggests that it does hold over the whole growth range (Raznahan et al., 2012), while other evidence suggests an inverse-U or J-shape relationship between fetal brain growth and later-life functioning (Schlotz and Phillips, 2009). Therefore, it is essential to study fetal brain development to further understand human development. This is one of the aims of the Utrecht YOUth cohort, a Dutch population cohort in which normal child development is being assessed from the fetal stage to adolescence (Onland-Moret et al., 2020).

One brain measure that correlates with several adversarial conditions in childhood and adulthood is the intracranial volume (ICV). It can be defined as the volume of the central nervous system below the cranium without the spinal cord or as the total volume of brain cellular mass and cerebrospinal fluid (CSF) together. It is known that the ICV correlates, on the genetic level, with the diagnosis of several psychiatric conditions such as schizophrenia (Smeland et al., 2017), bipolar disorder (Hulshoff Pol et al., 2012), and attention deficit hyperactivity disorder (ADHD) (Klein et al., 2019). Furthermore, it was also suggested that extreme environmental conditions during the prenatal period influence the ICV in adulthood (Hulshoff Pol et al., 2000).

A primary tool for assessing the fetal brain is ultrasound imaging (Monteagudo and Timor-Tritsch, 2009, 2012). Ultrasound has few safety concerns (O'Brien, 1998; Hata et al., 2010; Abramowicz, 2013), is relatively cheap, and its usage is easy for both the pregnant woman and the operator. Therefore, ultrasound is routinely used to assess almost every pregnancy in developed countries (Caradeux et al., 2019). One of the inherent challenges of ultrasound is its relatively low resolution and contrast created, for example, by fetal movements, amount of amniotic fluid, and shadowing by the ultrasound's beam interaction with the skull.

Today, the standard way of assessing brain developmental measures is still two-dimensional (2D) ultrasound imaging (Lin et al., 2019). However, since its introduction, three-dimensional (3D) or even 4D ultrasound imaging has attracted growing interest in diagnostics (Hata et al., 2010; Salman et al., 2011; Tonni et al., 2015). For example, 3D ultrasound can help locate the mid-sagittal plane and measure auxiliary brain structures (Dückelmann and Kalache, 2010). Beyond its diagnostic value, there is also a growing interest in 3D ultrasound for research purposes (Gonçalves, 2016).

One way to measure brain characteristics such as the ICV in 3D ultrasound imaging is through manual tracing (Albers et al., 2018). Indeed, many studies used the Virtual Organ Computer-Aided-Analysis (VOCAL) method to measure the volume of various brain structures (Rutten et al., 2009; Rizzo et al., 2012; Caetano et al., 2015; Babucci et al., 2019), even during the first trimester (Tonni et al., 2015). For large structures, like the ICV, VOCAL was proven to be a reliable method (Martins and Nastri, 2014). However, VOCAL is labor-intensive, time-consuming, and requires extensive training. Moreover, there might be some systematic bias even in cases where the inter and intra-observer intraclass correlation coefficient is high. Hence, for large cohorts like the one we are aiming to measure, VOCAL is not a feasible research solution. Instead, one would like to have an automatic computational-based method. Here, we compare an automatic method of ICV measurement to the manual tracing of ICV based on the VOCAL method. Later, we plan to use the automatic method in the context of the Utrecht YOUth cohort for assessing relationship between fetal growth and child development.

When developing and validating an automatic computationally-based algorithm for extracting or measuring a specific brain structure from ultrasound scans, one has to choose between traditional computational methods and the more modern machine-learning algorithms. It is pretty clear that the current emphasis among neuroimaging and computer scientists in the ultrasound field tends toward the machine-learning side (see for example, Namburete et al., 2018; Moser et al., 2022). Nevertheless, despite the current focus in ultrasound research on machine-learning algorithms, which hold great promise for image segmentation in clinical and research settings, the question remains whether more traditional computational methods should also find their place in the Swiss knife toolbox available for the research community. Why? On the one hand, in the field of magnetic resonance imaging (MRI), registration and segmentation tools are still widely used in clinical and research contexts (Nerland et al., 2022). On the other hand, as noted above, ultrasound imaging analysis confronts fundamental challenges associated with image quality.

Recently, we have developed a registration-based pipeline to measure longitudinal ICV changes during aging using MRI (Caspi et al., 2020). This pipeline was especially good at identifying minimal ICV changes during aging, which probed us to ask whether it can also be utilized for ICV extraction during development. Note that applying such an MRI-tested tool to fetal ultrasound modality is not necessarily straightforward due to the differences in image quality between these two modalities. The pipeline registers the brain of a participant to an average brain model and calculates the ICV of the subject from the inverse of the final transformation and an ICV mask of the average brain model. We anticipated that the same procedure would enable us to measure the ICV in 3D fetal ultrasound images.

By comparing the results of the automatic ICV measurements to those of a manual ICV tracing on fetuses at gestational ages (GA) of 20 and 30 weeks, we show that the automatic intensity-based registration procedure for ICV measurements was highly consistent with the results of manual ICV measurements using the VOCAL method. Moreover, our approach reproduced both the non-linear pattern of fetal brain growth and the increased inter-subject variability in ICV values in later stages of fetal development. However, unlike the manual measurements, the automatic method did not provide sufficient statistical evidence for ICV sexual dimorphism at a GA of 30 weeks.

## 2. Methods

### 2.1. Cohort description and scan acquisition

The 3D ultrasound scans were acquired within the YOUth Baby and Child cohort (Onland-Moret et al., 2020). In this cohort, up to 3,000 individuals are being followed from the fetal stage to childhood, including using fetal ultrasound around a gestational age (GA) of 20 weeks and again around 30 weeks. From this dataset, we chose at random a subset for the validation of our automatic measurements (approximately 100 subjects in each age group). The scans for this subset were randomly selected by the data manager of YOUth cohort. Regular quality checks are performed to ensure the overall high-quality standards of the ongoing YOUth cohort study and are available for usage by researchers associated with the cohort. The total number of scans was selected based on the work time available to the human rater that measured the ICV manually as part of her research activities (see below). We analyzed 92 3D ultrasound scans (42 females) at GA of 140–170 days (mean GA 152.9 days). For the older age group, we analyzed 90 ultrasound scans (39 females) with GA of 203–230 days (mean GA of 213.6 days). Below, we refer to these two groups as 20- and 30- weeks groups. Thus, altogether our dataset included 98 unique fetuses (43 females). For 84 cases (38 females), we had the measurements of the same fetus at two different GA. The full statistical description is shown in Supplementary Table S1.

For a complete description of the ultrasound scan acquisition, (see Albers et al., 2018). In short, 3D ultrasound images were acquired transabdominal using a Voluson E10 (GE Healthcare, Zipf, Austria) ultrasound machine with a 2–6 MHz convex probe (RM6C). Overall, ten experienced sonographers participated in data collection as part of the YOUth cohort. To acquire 3D-ultrasound images, a sweep angle of 65° was used, which covers the entire fetal skull. After the acquisition, the images were transferred to an offline cluster that contained the GE Healthcare 4DView program.

For each fetus in each age group, several ultrasound images were available from the YOUth cohort as were collected for the cohort purposes independent of this research. For the automatic and manual measurements of the ICV, the highest quality ultrasound image for each subject was chosen for further analysis by two authors (RL and IJI). This procedure was carried out separately for the automatic and manual measurements. In both cases, the researchers selected the scans blind to sex, any other identification information, or any information concerning functioning in later life.

### 2.2. Manual segmentation

All manual segmentations were done by one of the authors (RL), see further details in the Supplementary material.

We compared the manual ICV traced mask results to the automatic ones (see below) by calculating the linear fit R^2^ of the two implementations of our method (see the Results Section). Unfortunately, we could not calculate other standard measures of comparison between two binary masks, such as the Dice Similarity Coefficient and the Centroid Distance. The reason is related to the way the VOCAL program exports the manually traced ICV masks. 4DView exports the ICV masks in the form of an stl mesh file. However, this mesh does not align with the original ultrasound scan after conversion to a Neuroimaging Informatics Technology Initiative (nifti) format, for example, using the program 3D Slicer. Aligning the manually-traced nifti converted ICV mask to the original brain necessitates a tedious manual procedure and is never perfect (see Section 2.3.5 below for an example where such a procedure was followed). Hence, we could not compare the manual and automatic ICV masks in terms of the measures mentioned above. Note that this deficiency of calculated comparison measures lays fully outside our automatic developed method and our control.

### 2.3. Automatic segmentation

The procedure for obtaining an automatically segmented ICV mask is described below. In several steps related to the development of the pipelines, we had to make some practical choices concerning the parameters to use or the preparatory steps to apply. In these cases, the choices were based on either pre-acquaintanceship with the behavior of the algorithm (see Caspi et al., 2020; Buimer et al., 2021) or rational choice followed by an empirical test based on general principles of image analysis (e.g., steps taken during the ultrasound scans export).

#### 2.3.1. Ultrasound scans export

Ultrasound scans were exported from the manufacturer format (.vol) to Digital Imaging and Communications in Medicine (Dicom) using the 4DView program. Next, the Dicom images were converted to nifti format using the command dcm2niix from MRIcron (https://www.nitrc.org/projects/mricron/). Given that we used an intensity-based algorithm, which is sensitive to sharp edges, we decided to reduce the sharpness of the sweep boundary between the sweep field of view and its borders in standard ultrasound imaging. In particular, this was done by a self-written Python script that replaces all the voxels with a value of zero by the average grayscale in the scan. The effect of this procedure is that black voxels are replaced by gray ones.

After exporting the .vol files to Dicom, we noticed that two head orientations appear in each age group. Moreover, in some cases, the conversion to Dicom did not work correctly in the sense that the converted scans were mirrored in various planes to the orientation at which they should have been. In those cases, we used a self-written script in Python to rotate the images back to one of the two primary head orientations.

#### 2.3.2. Automatic ICV measurements

To measure fetal ICV automatically, we used a monomodal ultrasound registration algorithm between the fetal brain and a brain model (see below). Our method is an extension of the method published in Caspi et al. (2020) and further used in Buimer et al. (2021) for magnetic resonance images. In previous versions of this algorithm, we have used a pipeline that is based on the Minc-toolkit (Medical Imaging NetCDF Toolkit) (Vincent et al., 2016). Here, we used version 1.9.16 of the Minc-toolkit. The Minc registration was controlled by standard parameters of the Minc-toolkit (see Supplementary material). In addition, we extended the algorithm by implementing it in a separate pipeline based on the more modern registration package of Elastix (Klein et al., 2010; Shamonin, 2013). The Elastix registration steps were controlled by standard parameters of the Elastix package (see Supplementary material). Elastix version 5.0 was used for the majority of this work. However, for an unknown reason, the scaling plugin of Elastix (SimilarityTransform) that includes translation, rotation, and constant scaling did not compile on our machine. Therefore, Elastix version 4.8 was used when global scaling was needed during the pipeline run. In principle, we used the SimilarityTransform only for cases where the default algorithm during the rigid registration step that was based on the EulerTransform (translation and rotation) did not show sufficiently good results based on the quality control criteria discussed below. These cases mainly include subjects with a relatively significant total ICV difference from the model one.

The rationale for using two different implementations, i.e., Minc and Elastix, is to achieve two independent automatic measurements. Registration procedures are based on specific registration metrics, registration optimizers, and steps such as blurring or down-sampling (Che et al., 2017). By using two different implementations with different optimizers and optimization metrics, we increased the probability of independence. Note that the two pipelines were not combined during the computational steps. Only the final ICV value was calculated from the average value of the two pipelines.

The Minc-Toolkit pipeline was implemented in C++ and then warped in a python script written within the fastr environment for pipeline development (Achterberg et al., 2016). By contrast, the Elastix-based pipeline was implemented directly in the fastr environment. All computations were carried out on the Utrecht High-Performance Cluster (HPC).

**Figure 1:**
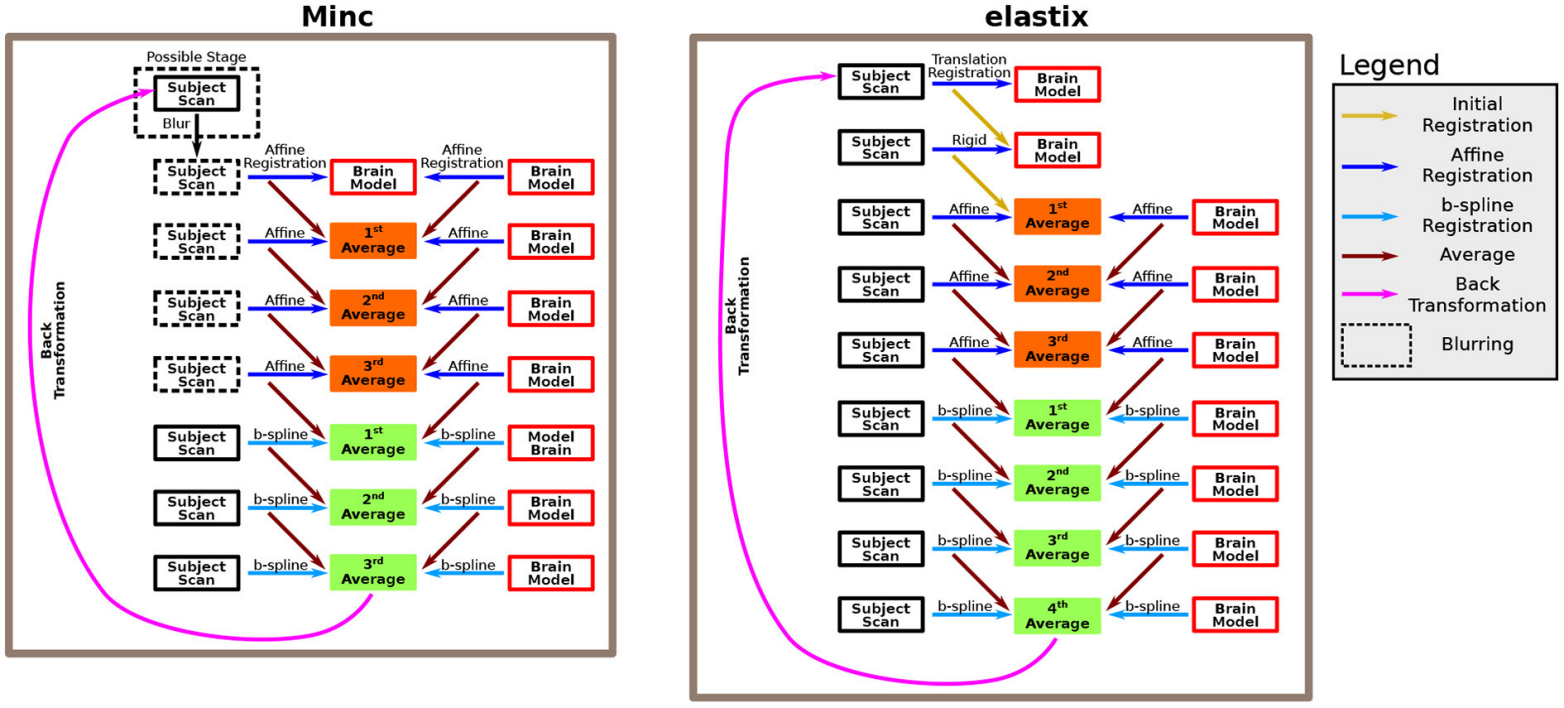
Description of the algorithm. The algorithm is based on several consecutive registration steps between a brain model and a subject brain. We used both Minc-based registration (left) and Elastix-based registration (right). For the Minc-based case, a sequence of four “affine” transformations followed each other. The “affine” registration steps include, in addition to standard affine transformation, both translation and rotation. At each stage, both the brain model and the subject scan are registered to an averaged image created in the previous step. In the first step, the brain model serves both as the registration target and one of the scans that are being registered. Moreover, an initial blurring step can be included. In that case, for all the “affine” registrations, the images are blurred. Subsequently, three B-spline registrations are applied using the same rationale. For the Elastix-based case, the subject scan is first registered to the brain model using a translation-based step. Next, a rotation (or rotation+ global scaling) is applied. Subsequently, the same algorithm as in the Minc-based case is applied. However, in that case, four B-spline registrations were used. For a complete description of the pipelines, see the Methods Section.

In both cases, the pipelines use several consecutive intensity-based registration steps to obtain a good registration match between two scans. In particular, it registers a brain model and a subject's ultrasound scan together. The brain model is accompanied by an ICV mask, which was drawn manually (see below). The two pipelines measure the ICV of the subject by calculating the reversed transformation of the final registration step, applying it to the brain model ICV mask, and counting the number of voxels in the newly formed ICV mask of the subject. A recap of the two algorithms used by these two pipelines is shown in Figure 1.

All codes are freely available from the authors upon request.

#### 2.3.3. Registration algorithm

The registration algorithm is based on a series of rigid, affine, and B-spline refined registrations between a fetal brain scan and a brain model. After each of the registration steps, an average image is calculated between the registration results of the two scans. This average image is used as the registration template at the following stage. For the Minc-Toolkit pipeline. the first affine registration used the brain model both as the registration template as well as one of the scans that are registered. Note that although many registration procedures recommend down-sampling to obtain better registration results of ultrasound images (Pratikakis et al., 2003), we did not use down-sampling in our pipelines. For specific parameters used for the application of the two pipelines, see Supplementary material.

After the last registration steps, the pipelines calculated the inverse transformation from the brain model to the subject's scan and applied it to the ICV mask of the brain model. Finally, the total ICV of each subject was calculated based on the total number of voxels in the newly formed subject's ICV mask multiplied by the volume of one voxel.

Note that we needed to change some registration parameters to achieve a successful registration in some cases. For the Minc-based registration package, this included adding the pre-blurring before the affine registrations. The Elastix registration package has many control parameters. Most of the time, the parameters that had to be tweaked were (i) changes in the optimizer between the automatic optimizer AdaptiveStochasticGradientDescent and the standard one (StandardGradientDescent); (ii) for the standard one, the value of parameter SP_a had also sometimes to be adjusted; and (iii) the ImagePyramidSchedule, which controls blurring (pyramids) during registration, was also sometimes adjusted. In general, only the parameters for the translation, rigid, and first and second affine transformations were tweaked.

#### 2.3.4. Brain models

We included a subset of the ultrasound scans from the validation cohort used in this work to construct the brain models. The criterion for inclusion of a subject in the calculation of the average brain model was that its ultrasound scan had a relatively high quality compared to the rest of the subjects as it was judged based on personal impression (judged by IJI). These ultrasound scans were used to calculate a brain model using the Minc-based pipeline. The algorithm for constructing the brain model is almost identical to the one for calculating the ICV masks. The primary differences are: (i) at the first stage, one of the subjects used for constructing the brain model is used as a template for registration; (ii) at each step of the algorithm, all subjects included in the brain model construction were registered to the average registration result of the previous step; (iii) four B-spline steps were used instead of three; and (iv) instead of calculating the inverse transformation, the brain model output was the average of all registered subjects after the fourth B-spline registration step.

Using the intensity-based registration algorithm to calculate the ICV worked best when there were no significant size differences between the subject brain and the brain model. Since the brain grows substantially during fetal development, we have used different brain models for the two different age groups, i.e., 20 and 30 weeks of GA, respectively. In addition, as stated above, two head orientations were identified after exporting the ultrasound scans to nifti. However, instead of mirroring the images of one of these two groups to the orientation of the other, we decided to use two different brain models for each age group. The rationale was that we wanted to interfere as little as possible with pre-processing steps. We refer to them as the “Left” group and the “Right” group. Thus, we ended up with four brain models, two for the 20 weeks age group and two for the 30 weeks age group. For each of these four brain models, one of the authors (IJI) edited an ICV mask by manually controlling which voxels should be considered part of the ICV and which lay outside the ICV region.

We averaged 13 ultrasound images for constructing each of the two brain models at GA of 20 weeks. For the GA of 30 weeks, we used 11 and 17 ultrasound scans for the “Left” and “Right” groups, respectively. The number of subjects used for constructing the brain models was based on a rule of thumb. It took into account both the theoretical need to average a relatively large number of subjects to obtain good quality brain model and the practical limitation of computer time needed for the averaging. For three out of the four groups, choosing 10–20 subjects from each group for averaging resulted in a good quality brain model after one trial round. However, for the “Left” group of the GA of 30 weeks, we had to repeat the attempts to create a brain model several times (14–11 subjects) until we obtained a good enough quality of the brain model where the typical brain structures were clearly identified.

#### 2.3.5. Mask development

Several steps were implemented to create an accompanying ICV mask for each one of the four brain models. First, we chose four ultrasound scans as templates. We used the manually delineated ICV mask for each of these subjects and exported them from 4DView to an stl mesh file. Next, we used a python script within the program Slicer (https://www.slicer.org/) to manually match, as much as possible, the stl ICV mesh file to the nifti ultrasound file previously created for this subject. The matching was done using a series of translation and rotation steps. Subsequently, the ICV mash file was converted to a nifti format. Next, these subjects and their accompanied ICV masks were used as models, and our Minc-based pipeline registered the calculated brain models to the four subjects, respectively, to create the brain models that approximate the ICV masks.

To obtain the highest quality ICV masks for the brain models, one of the authors (IJI) manually edited the four ICV masks based on the anatomical knowledge of the fetal brain. The procedure for editing the ICV masks included adding voxels in the ICV mask (especially around the edges) that are anatomically supposed to be included in ICV masks and erasing parts that were wrongly included in the tentative ICV masks. This procedure was repeated for all three projections (axial, coronal, and sagittal) and for all planes until the ICV mask was considered anatomical adequate. Finally, a median filter with a kernel of three voxels was applied three times to smooth the ICV mask to an appropriate level.

In practice, the segmented ICV includes the total sum volumes of the brain cellular mass and CSF. Note that the skull base shadows the brainstem in an axial projection of ultrasound scans but less so the cerebellum (Pilu et al., 2007). Thus, though we included the cerebellum in our mask, we cannot know what portion of the brainstem is included in our measurement.

#### 2.3.6. Quality controls

When measuring structures automatically from biomedical images, one is constantly faced with the problem that the ground truth is unknown. Hence, artifacts and false measurements can occur. We sought a procedure for making an automatic distinction between measurements that have a high probability of representing the actual ICV and those that do not. By implementing our intensity-based registration pipeline with two different computational registration packages, we could construct a well-defined criterion for a distinction between these two cases. The rationale behind this approach is that the parameter space of two independent registration packages should be somewhat decoupled. Hence, errors that occur in one of the cases would probably not occur in the other.

Consequently, quality control (QC) for the automatic ICV calculation was based on the standard deviation (SD) between the Elastix-based and Minc-based pipelines ICV calculation. Empirically, as further discussed in the Results Section, we showed that an SD of about 10% of the ICV for either age groups was a suitable threshold for deciding which subjects can be considered outliers and, therefore, excluded from the analysis.

In practice, we repeated the registration for subjects with an ICV SD larger than the threshold (using the Minc-based pipeline, the Elastix-based pipeline, or both) with different registration parameters. If, after one or two rounds of parameters tweaking, the SD of the ICV was still above the threshold, we excluded this subject from further analysis. To assess which of the two pipelines was responsible for the large SD, we overlaid a picture of the ICV outline for several planes over the ultrasound scan image. These images are produced automatically by the two pipelines. This procedure was done blinded to participant information such as gender.

For the Elastix-based pipeline, we added an additional QC step. This test was based on calculating a similarity grayscale metric between the subject scan and the registration average at the last stage of the non-linear registration. From the many possible similarity metrics that exist (Che et al., 2017), we used for the calculation:


$$Similarity=\frac{\underset{i,j,k}{\sum }SubjectScan_{ijk}\times Average_{ijk}}{{\left(\underset{i,j,k}{\sum }SubjectScan_{ijk}^{2}\times \underset{i,j,k}{\sum }Average_{ijk}^{2}\right)}^{0.5}}$$

Here, *SubjectScan*_*ijk*_ is the grayscale value at voxel with indices i,j, and k of the subjects scan and similarly for *Average*_*ijk*_. The value of this similarity can vary between 0 (no overlap) and 1 (full overlap). *Post-hoc* analysis suggested that a value below 0.7 is too low and indicated a wrong ICV registration.

### 2.4. Statistical analysis

Data analysis was performed using the statistical computing environment R (Team, 2020). Intraclass Correlation Coefficients were calculated using the R *psych* package. Graphs were plotted using the *ggplot2* package.

### 2.5. Image preparations

All images were prepared using Inkscape (RRID:SCR_014479) and GIMP (RRID:SCR_003182).

## 3. Results

### 3.1. Brain models

**Figure 2:**
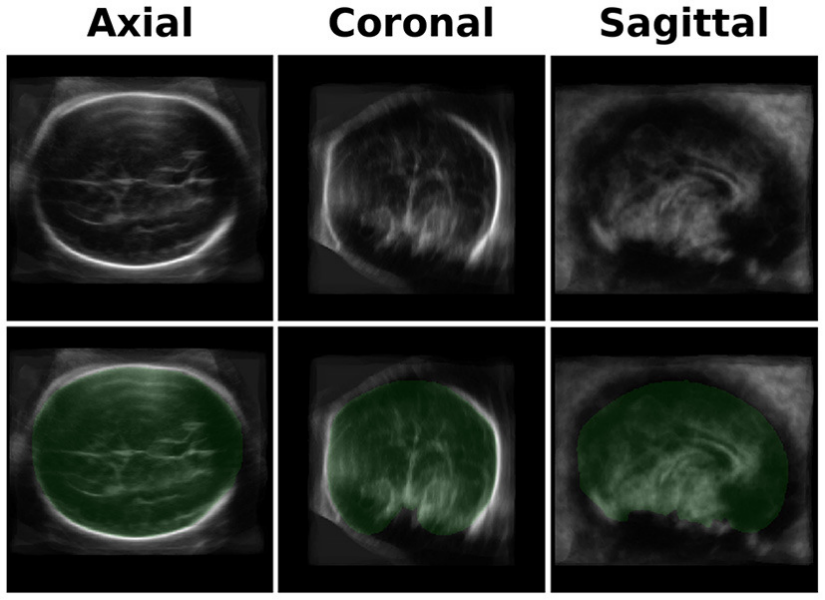
Brain Model and ICV Mask. Upper row-an example of three projections (axial, coronal, and sagittal) of one of the brain models used for the registration. The current one is one of the two models used for the GA of 30 weeks group. Lower row - a mask (in green) of the manually traced ICV mask of the brain model.

We developed an averaged brain model for both the 20- and 30-week GA groups by averaging the brains of 10-17 subjects using the same pipeline used for the ICV calculation as described in Methods Section. An example of the axial, coronal, and sagittal planes of one of the brain models is shown in the upper row of Figure 2.

An example of the three-axis projection of one ICV mask is shown in the lower row of Figure 2. The four ICV masks (for the two age groups and the two head orientations) were used for calculating the subjects' ICV based on the inverse registration of the brain models and the subject brain images.

### 3.2. Validation of the automatic measurement results

**Figure 3:**
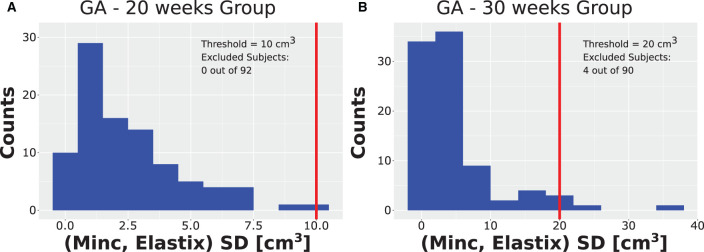
Distribution of SD. Distribution of the SD ICV values between the Elastix-based and Minc-based pipelines for **(A)** the group with GA of 20 weeks and **(B)** the group with GA of 30 weeks. Note the long tail distribution in both cases. Vertical red lines represent the threshold chosen for the rejection of outliers. Note that for the GA of 20 weeks, the threshold chosen was ten cm^3^. Due to binning presentation, the case with the largest SD is grouped together with the threshold despite being smaller. Legend inside each panel provides details about the threshold and number of subjects rejected from the analysis for each age group.

After running the registration algorithm twice, once based on the Minc-toolkit registration package and once based on the Elastix registration package, we noted a long-tail distribution for the calculated standard deviation (SD) between the results of these two computational packages. The results of the SD distributions are shown in Figure 3A for GA of 20 weeks and in Figure 3B for GA of 30 weeks. At GA of 20 weeks, the SD was below 10 cm^3^ for all cases. A manual inspection of the outline of the generated ICV over the subjects' brain images suggested that even for the cases with the largest SD, it was hard for a human rater to decide which of the two automatically calculated ICV masks was more closely representative of the ground truth. Hence, we decided not to exclude any subject from further analysis. For the GA of 30 weeks, the SD distribution also had a long tail. However, in that case, the tail was much longer as compared to the 20 weeks group. Moreover, some results did not fit well with the individual ICV, as judged by the human rater (IJI). Hence, we set a criterion of 20 cm^3^ for the exclusion of subjects from further analysis. Individuals with ICV SD above this value were excluded from further analysis. Though this criterion is somewhat arbitrary, it was based on the inspection of the SD distributions (Figure 3A) and choosing a value that removes most of the long tail part of the distribution while maintaining the largest number of subjects included in the final analysis. We also tried to analyze the data with a somewhat different exclusion criterion (e.g., 15 cm^3^). However, since this change did not substantially influence the final results, we report here only the results with an exclusion criterion of 20 cm^3^. Overall, four subjects (4.4%) were excluded based on this criterion. All the excluded subjects were males. We do not know what is the source of this gender imbalance. Based on the average ICV of subjects (84 ± 16 cm^3^, 276 ± 37 cm^3^), the exclusion criterion should probably be somewhere between 7.5% and 12% of the average ICV for future usage of our procedure.

**Figure 4:**
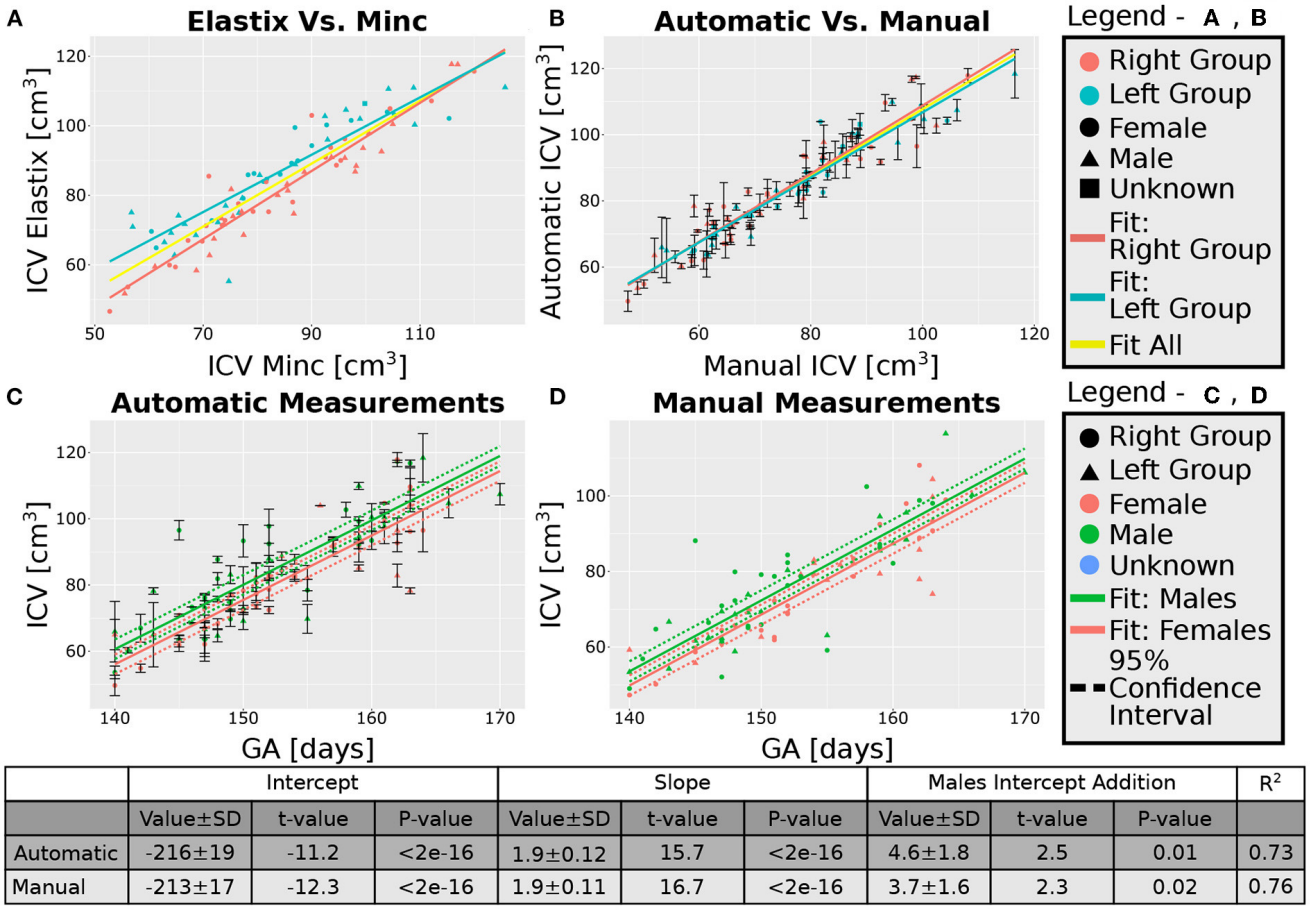
Analysis of the group with GA of 20 weeks. **(A)** Correlation between the results of the two pipelines (Elastix- and Minc- based). **(B)** Correlation between the automatically calculated ICV and the manually traced ones. Error bars are the SD of the Minc- and Elastix- based results. For **(A,B)**, the three lines represent linear fits of the data for the “Left” group (light blue), “Right” group (pink), and for all subjects together (yellow). For details of the fits, (see Supplementary Tables S2, S3). **(C,D)**—ICV as a function of GA for **(C)** automatically calculated results and **(D)** manually traced ICV. For **(C,D)** pink solid line—fit of the data for females, solid green line—fit of the data for males. Dashed lines represent the 95% confidence intervals of the intercept difference for males and females. The table below the graphs provides details about the fitting results of the ICV as a function of GA with intercept-dependent sex.

**Figure 5:**
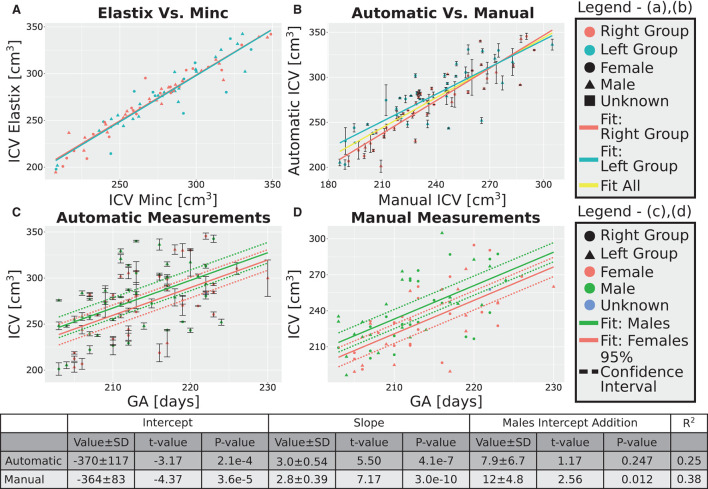
Analysis of the group with GA of 30 weeks. **(A)** Correlation between the results of the two pipelines (Elastix- and Minc- based). **(B)** Correlation between the automatically calculated ICV and the manually traced ones. Error bars are the SD of the Minc- and Elastix- based results. For **(A,B)**, the three lines represent linear fits of the data for the “Left” group (light blue), “Right” group (pink), and for all subjects together (yellow). For details of the fits, (see Supplementary Tables S2, S3). **(C,D)**—ICV as a function of GA for **(C)** automatically calculated results and **(D)** manually traced ICV. For **(C,D)** pink solid line—fit of the data for females, solid green line—fit of the data for males. Dashed lines represent the 95% confidence intervals of the intercept difference for males and females. The table below the graphs provides details about the fitting results of the ICV as a function of GA with intercept-dependent sex.

After excluding the four subjects from the 30 weeks GA group, we obtained an overall good agreement between the results of the Elastix-based registration pipeline and the Minc-toolkit-based one. The results are shown in Figures 4A, 5A. Full parameters of the fits can be found in Supplementary Table S2. The coefficient of determination (R^2^) for the linear fit between these two calculations was 0.84 and 0.90 for the 20 and 30 weeks GA groups. For further analysis, we have used the average calculated ICV between the two registration package pipelines as the value for the automatically calculated ICV. Using the average ICV of these two intensity-based registration pipelines, we found a high degree of correlation between the manual measurements of the ICV, as was delineated by one of the authors (RL), and the automatic ones. For the 20 weeks GA group, the results are shown in Figure 4B. For the 30 weeks GA group, the results are shown in Figure 5B. Full parameters of the fits can be found in Supplementary Table S3. The linear fit R^2^ was equal to 0.90 and 0.76 for the 20- and 30- weeks GA groups, respectively. However, for both age groups, the absolute value of the automatically calculated ICV was larger than the manually calculated one (84 ± 16 vs. 76 ± 15 cm^3^; and 274 ± 35 vs. 237 ± 28 cm^3^). We believe that the reason for this discrepancy is related to the hindbrain delineation. The hindbrain is better seen in the average brain models than in the images of the individual subjects. Hence, it might have been better represented in the average brain models and therefore in the automatically generated ICV compared to the manual measured ICV. Nevertheless, the linear relationship between the manual and automatic measurements of ICV provides substantial support for the validity of the automatic procedure.

**Figure 6:**
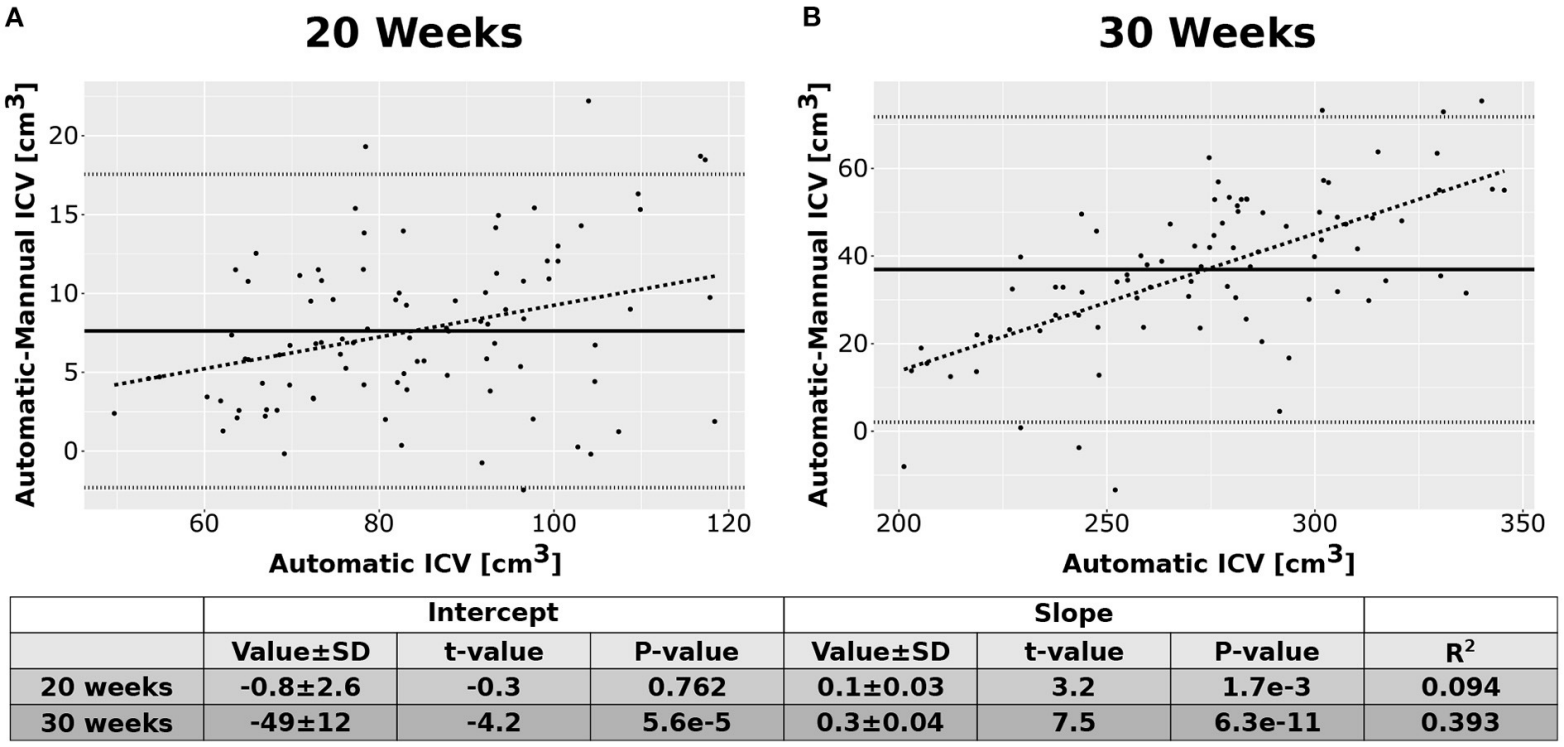
Difference between the automatic and manual methods. Difference between the results of the automatic ICV measurements and the manual ICV measurements as a function of the automatically calculated ICV. **(A)** Results for the group of fetuses at GA of 20 weeks. **(B)** Results for the group of fetuses at GA of 30 weeks. Solid lines—mean difference. Dotted lines—95% confidence intervals base on t-statistics. Dashed lines—linear fit of the difference as a function of the automatically calculated ICV. The tables below the graphs provide details about the fitting results.

As an additional layer of control, we calculated the Intraclass Correlation Coefficients (ICC) for the automatic and manual measurements of the ICV. Overall, the ICCs were between good to excellent (Shrout and Fleiss, 1979). For the 20 weeks GA group, we report an ICC of 0.95 (bounds-0.93, 0.96) for the manual rater and 0.97 (0.96, 0.98) for the average of the automatic and manual rating. For the 30 weeks GA group, we report an ICC of 0.85 (bounds-0.79, 0.89) for the manual rater and 0.92 (0.88, 0.94) for the average of the automatic and manual rating. Note, however, that we have noticed some bias in the difference between the ICV calculated by the manual rater and the automatically calculated one (Figure 6). This bias was manifested in a linear relationship between the automatic and manual ICV difference and the value of the ICV itself. We do not know if the source of this bias is related to the manual delineation of the ICV or the automatic calculation.

### 3.3. Comparison of the “Left” and “Right” groups

One caveat of our procedure might be the usage of two different models for each age group, as discussed above. Altogether, in our dataset, we had 42 ultrasound scans (18 females) in the “Left” group and 50 ultrasound scans (24 females) in the “Right” group for the 20 weeks GA group. Similarly, we had 39 ultrasound scans (15 females) in the “Left” group and 47 ultrasound scans (24 females) in the “Right” group that passed our QC for the 30 weeks GA group. To check the possibility that this choice potentially created a biased in our calculation, we evaluated the distribution of ICV values obtained from the two models for each age group. The results are shown in Supplementary Figure S2. The calculated *t*-test *P*-values for comparing the two brain models (abbreviated by us as “Left” and “Right”) were 0.47 and 0.48 for the 20- and 30-weeks GA groups. Thus, we concluded that working with two brain models for each age group did not bias our results. Consequently, we report the results of the “Left” and “Right” groups together in the following analysis.

### 3.4. ICV and age correlation

A linear relationship was found between the automatic ICV measurements and GA for each age group separately (Figures 4C, 5C). Similar linear relationships between the ICV and GA for the manual cases are shown in Figures 4D, 5D. Detailed statistical characteristics of the fitting for both the automatic and manual measurements of the ICV are shown in the tables in Figures 4, 5. As can be seen, the automatic and manual results corresponded well to each other in terms of the linear relationship between ICV and GA. This fact provides additional support for the validity of the automatic procedure.

### 3.5. ICV and sexual dimorphism

We used sexual dimorphism, the difference in the average ICV between females and males, as an additional test for the validity of the automatic extraction method. Detection of small group differences between different groups, if they exist, can increase trust in an automated extraction method. However, despite the general correspondence between the automatic and manual measurements of the ICV, there was one crucial difference between these two approaches concerning ICV sexual dimorphism. While sex differences in the ICV with a *P*-value below 0.05 were found within the automatic and manual measurements for the younger age group, it was not the case for the ICV results at 30 weeks GA (Figures 4C,D, 5C,D). For the older age group, adding sex as a covariate to the linear model of the manual measurements resulted in a *P*-value of 0.01 for the sex label. By contrast, for the automatic measurements, the *P*-value for the sex label was 0.25. The 95% confident intervals for each approach are shown in Figures 5C,D as dashed lines. As can be seen, the findings of the manual measurements suggest a statistically-based sexual dimorphism around 30 weeks of GA. By contrast, the automatic measurements did not provide support for such a conclusion, or they might suggest that the effect is too small to be detected within our limited sample. Note, however, that in both the manual and automatic approach, males had larger ICV than females, as is evident from the fitting lines of the two sex groups.

### 3.6. ICV growth rate

**Figure 7:**
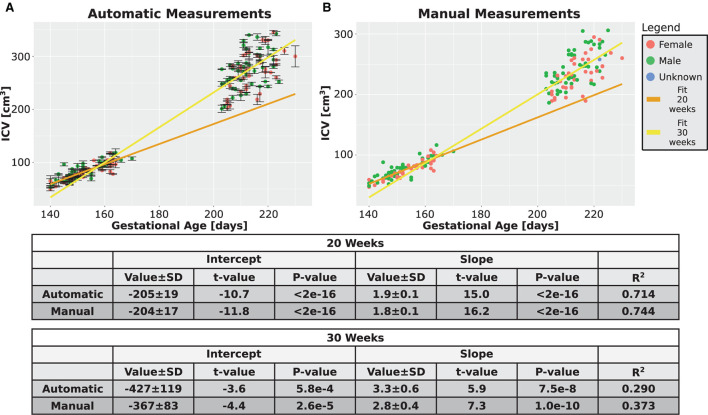
Combined time points graphs. Reproduction of the results from Figures 4C,D, 5C,D of the two age groups only for the subjects that had two measurements. **(A)** Automatically calculated ICV. **(B)** Manually traced ICV. Dark and light yellow lines are fits of the two age groups without differentiating between females and males. The tables below the graphs provide details about the fitting results.

Next, we checked whether additional support for the validity of our automatic procedure could be obtained by studying the growth behavior of the ICV. The growth graphs are shown in Figure 7A for the automatic method and in Figure 7B for the manual method. Linear fits for the relationship between ICV and GA are also shown in the same figure. Three observations can be deduced from the two panels of the figure. First, qualitatively, the automatic and manual measurements showed the same behavior. Second, in both cases, the slope of ICV GA dependency is steeper for fetuses at an older age. This finding suggests that there is an accelerated growth of the brain volume as the fetuses develop. The same fact can also be seen from the relationship between the GA at which a subject was first scanned and the ICV difference per day for that individual (see Supplementary Figures S3A,B for the automatic and manual measurements). Third, as is evident from Figure 7, the intraindividual variability increases for older GA for both the automatic and the manual measurements. Although we did not directly quantify this observation, this observation provides additional support to the validity of the automatic procedure to calculate the ICV.

To summarize all the findings of the Results Section: Based on this analysis, we concluded that the automatic procedure for calculating fetal ICV, as is presented here, is a valid way to measure the ICV.

## 4. Discussion

An important research question is how normal fetal brain development correlates with future cognitive and behavioral outcomes. Automatic computational-based methods are paramount for these efforts. Many times, extracting the brain or the intracranial volume is the first step in downstream automatic analysis pipelines. Over the years, multiple computational-based methods have been developed for detecting, classifying, segmenting, and registering ultrasound scans (e.g., see Kutarnia and Pedersen, 2015). For detailed methodological reviews for ultrasound registration or segmentation of global shape and internal structures and features, (see Che et al., 2017 and Torres et al., 2022). See also references therein for a complete list of methods previously used. In principle, one can divide registration-based and segmentation methods into those based on intensity similarity and those using feature selection. Initial attempts to register 2D ultrasound images were intensity-based and applied several filtering methods to reduce the inherent limitations of ultrasound images (Cen et al., 2004). More modern attempts to register or align 2D ultrasound scans are based on artificial intelligence (e.g., Yaqub et al., 2015) or, in particular, on deep learning algorithms (Liu et al., 2019). For example, an automatic method was developed to measure fetal head circumference based on a Haal-like feature classifier, Hough transform, and ellipse fitting (van den Heuvel et al., 2018). Another study used a deep learning algorithm to measure brain circumference in real-life situations in under-developed countries (van den Heuvel et al., 2019). Similarly, a regional convolutional neural network was used to detect the feature of the standard axial plane for its identification (Lin et al., 2019). As a final example, a Bayesian Neural Network was used to estimate gestational age (GA) from an ellipse fitting of the skull (Lee et al., 2020).

Similar to 2D ultrasound, also for 3D ultrasound, initial registration attempts were intensity-based and used advanced filtering and down-sampling procedures (Pratikakis et al., 2003). Similarly, a Gabor transformation was used to identify the pose of fetuses based on the eyes' location (Chen et al., 2012). Followed steps that included rigid and non-rigid registrations to a reference model allow the measurements of several facial characteristics. Note that in this case, global skull features that are not necessarily available in all scanning settings were required. Moreover, a manual step of Region Of Interest selection was required for successful operation.

More recently, the field has moved from more traditional computational methods toward machine-learning algorithms for utilizing different aspects of ultrasound registration and alignment. For example, two-stage convolutional neural network (CNN) was used to obtain a full segmentation of the skull (Cerrolaza et al., 2018). In another example, a geometric-based feature detection using the point-drift method and random forest tree was used to register different scans of the same subject to each other (Perez-Gonzalez et al., 2020). Similarly, after manual brain editing, a constitutional regression network was used to estimate brain age based on its folding program (Namburete et al., 2017). In yet another example, A CNN network was used to align 2D planes to their corresponding 3D scan (Yeung et al., 2021). A similar approach utilized a multi-task, fully convoluted neural network to align a series of 3D ultrasound scans to a common space and extract the volume of the brain based on an ellipsoid fitting of the skull (Namburete et al., 2018). The main limitation of the Namburete et al. (2018) approach was that it disregards 3D information associated with the scan. Hence, as a continuation of that work, Moser et al. suggested an alternative CNN for extracting the brain from 3D ultrasound scans based on the complete 3D information (Moser et al., 2020). Finally, while working on this manuscript, the same group published an extension of their CNN algorithm and combined it with another machine-learning network for registration to a standard space (Moser et al., 2022). The main difference between the earlier (Moser et al., 2020) and latter (Moser et al., 2022) works concerning brain extraction is that the latter application includes an initial down-sampling and final up-sampling steps. Indeed, the latter work of Moser et al. showed excellent results and can deal with the internal limitation of fetal ultrasound imaging, such as increased ossification of the skull and position variations.

This work used the more traditional intensity-based registration procedure to analyze 3D ultrasound scans and extract the ICV. In principle, ICV is only one of the possible biomarkers that can connect fetal development and later life functioning. Indeed, traditionally medical imaging rely on a set of 2D measures such as the sizes of the lateral ventricles, cavum septi pellucidi, cisterna magna, and the corpus callosum (see Torres et al., 2022 and references therein). Also, the head circumference, biparietal diameter, and occipitofrontal diameter are widely used biomarkers to assess fetal growth (Monteagudo and Timor-Tritsch, 2012; Napolitano et al., 2020). Recently, machine-learning algorithms showed great promise for analyzing 3D structures in the fetal brain as possible biomarkers (Hesse et al., 2022). Here, we concentrate only on one crucial biomarker, namely ICV. We showed that our automated intensity-based registration and segmentation procedure could measure fetal ICV from 3D ultrasound images with high reliability and accuracy. Overall, once installed, the usage of the pipelines becomes quite an easy task, even for a non-experienced user.

We were able to reproduce two well-known characteristics of fetal brain growth, even with the relatively limited sample size we used to validate our method; Namely, we found (i) accelerated brain growth rate; and (ii) increased intrasubject variance of the ICV distribution size for older fetuses. Concerning these two findings, the automatic ICV measurements were consistent with the manual ones. In addition, we reported automatic and manual ICC values between good and excellent despite some bias observed in the measurement difference for smaller vs. larger ICV for each age group separately.

The fact that fetal brain growth is a non-linear process was shown multiple times over the years, especially during the late second and third trimesters of pregnancy (Roelfsema et al., 2004; Napolitano et al., 2020). Usually, a quadratic behavior is suggested for the non-linear dynamic (Hsu et al., 2013). For example, in an MRI study of fetal brain growth, a quadratic dependence on age was observed for various brain measures such as the head circumference and the skull biparietal diameter (Kyriakopoulou et al., 2016). However, in this study, the cortical volume showed exponential growth rather than a quadratic one. While, in contrast, in a relatively small sample size MRI study, no substantial statistical difference between the linear and quadratic models' description of the supratentorial volume growth was reported (Scott et al., 2011). We withheld from fitting an exact function for the ICV growth since we measured it only for two separate age groups and not throughout a continuous gestational age range. Thus, we cannot specify whether the ICV growth followed a quadratic, exponential, or any other functional form. Nevertheless, the fact that we obtained a non-linear growth behavior of fetal ICV supports our automatic method's validity.

In parallel to the observed non-linear behavior of fetal brain growth, the intraindividual variability also increased with GA, as was observed recently in the INTERGROWTH-21^*st*^ cohort (Napolitano et al., 2020). Similarly, an increased intrasubject variance was observed in another study for the thalamus, cerebellum, and cerebral cortex volumes (Babucci et al., 2019). Interestingly, in one study, the intrasubject variance increased with age for various volumetric measures but not for 2D measures like head circumference (Kyriakopoulou et al., 2016). In the current study, both the manual and the automatic measurements indicated an increased intrasubject variance for older fetuses. This finding provides support for the accuracy of the automatic procedure presented in this work.

In contrast with these two points discussed above, there was some disagreement about the ICV sexual dimorphism differences between the automatic and manual measurements at GA of 30 weeks. For the manual measurement, the difference between the ICV of the two sexes reached a statistical *P*-value smaller than 0.05. By contrast, the *P*-value was much higher for the automatic measurements, though males did have, on average, larger ICV than females. Whether the *P*-value will be above or below 0.05 depends, naturally, on the sample size and the effect size. Our sample size was quite limited since this study is a method validation study and not a detailed research study. Still, for the sake of validation, it is important to ask what the expected effect size might be and what the corresponding sample size should be to validate such an effect.

Previously, fetal sexual dimorphism differences were shown in several studies (e.g., Smulian et al., 1995; Rizzo et al., 2016). Moreover, it is well-known that the median head circumference of males at birth is larger than those of females (ratio of 1.032, see https://www.cdc.gov/growthcharts/clinical_charts.htm). Thus, the measured effect sizes of sexual dimorphism at birth are relatively small. In accordance with these findings, three studies estimated a ratio of 1.014 to 1.016 between the head circumference of males and females; (i) across the GA range of 20–37 weeks, (ii) at an average GA of 34.5 weeks, or (iii) average GA of 30 weeks (Schwärzler et al., 2004; Melamed et al., 2013; Yeo et al., 2017). Similarly, another study that used laser measurements of the outer cranial volume for newborn babies estimated an average ICV ratio of 1.066 and an average head circumference ratio of 1.02 between males and females (Vermeulen et al., 2021). Interestingly, in a large Dutch cohort, sexual dimorphism differences were observed already in the first trimester, and the measured head circumference for males was larger than that of females (Broere-Brown et al., 2016). However, in that study, the differences were significantly reduced from about 0.5 SD at GA of 20 weeks to about 0.35 SD at GA of 30 weeks. If indeed ICV sexual dimorphism decreases with GA, this fact might explain our findings and the difference between the consistency of the two measurement methods at a younger GA relative to their inconsistency at an older GA. Note, however, that some studies did not find sexual dimorphism differences for various brain measures (Kyriakopoulou et al., 2016; Kavak et al., 2021).

Thus, the published literature suggests that the ratio of the average fetal ICV for males and females will be somewhere between 1.02 and 1.05. Based on these values, the automatically measured average ICV for females, and its SD, one can calculate that a sample size between 550 and 96 is needed to detect such an effect with a type I error probability of 0.05 and a type II error probability of 0.8. Since the ground truth for the differences in the fetal ICV between females and males is unknown, the *P*-values that the automatic and manual methods reported concerning the fetal ICV sexual dimorphism are compatible with the statistical expectations. I.e., it might be the case that (i) the automatic method underestimated the difference; that (ii) the manual method over-estimated it; or (iii) both. Thus, we believe that our results suggest that the ICV of males is larger than that of females at GA of 30 weeks, but they are inconclusive concerning the size of the effect. Nevertheless, this discrepancy might suggest that the gold standard (manual measurements) against which we validated our automatic ICV extraction method might not be the best choice. Indeed, we consider, in future studies, validating the current automatic method against other available automatic methods.

One of the advantages of our method, and the fact that it uses two independent computational packages to calculate the same volume, is that it provides a simple criterion to differentiate between measurements that can be trusted and those that cannot. The criterion to differentiate between these two cases is based on the difference in the outcomes of the Minc and Elastix packages. When the difference of ICV between the packages is relatively large, the measurements cannot be trusted. When the difference is relatively small, the results of the automatic procedure can be trusted. Though the criterion we set for rejection of results is not based on a precise mathematical formula but rather on the impression from the results, we can suggest that deviation between the two automatic measurements of more than approximately 10% of the average ICV for subjects at a specific age group raises a red flag for the validity of the results. The percentage of images that had to be rejected remained below 5%.

Another advantage of our method is its usage of non-linear B-spline registration steps that make a local adjustment to the ICV mask. Usually, this step is still not used in machine learning algorithms, and the registration is restricted to translation, rotation, global scaling, and sometimes affine transformations (see, for example, Moser et al., 2022). This step is predicted to better fit local differences in the ICV of different individuals.

Despite our ability to validate our automatic procedure to measure the ICV from fetal 3D ultrasound scans, our procedure also has some limitations. First, the intensity-based method does not perform well if there is a large difference between the size of the brain model and the subject's ICV, for example, if the brain model is constructed from subjects with GA relatively different from the subject GA. Note that in the context of the YOUth cohort, fetal development is assessed around a GA of 20 and 30 weeks. During this period, the brain goes through major developmental changes. For example, many primary and secondary sulci and gyri start to emerge during this period (Stiles and Jernigan, 2010; Budday et al., 2015), though some major brain fissures can be detected by 2D ultrasound even before 20 weeks (Correa et al., 2006). Similarly, also the cerebellum experiences a period of accelerated growth, and its relative size relative to the telencephalon and diencephalon changes. As a result, the difference between the typical brain of fetuses at 20 weeks GA and the one at 30 weeks GA is not only the result of a scaling transformation. Consequently, we used separated brain models for the 20 weeks and 30 weeks groups. Based on the distribution of ages we examined for each age group (140–170 gestational days for the younger fetuses and 203–230 gestational days for the older ones—see Supplementary Table S1), it is safe to say that our method works well for model brains for every four gestational weeks. Models for a shorter period (e.g., two gestational weeks) will probably improve our observed bias (see Figure 6). Hence, although this is a limitation of our method, it is a limitation that could be expected from the need to register relatively similar model and subject brains.

A second limitation of our methods is that we have used two brain models for each age group. One of the limitations of transabdominal ultrasound imaging is the inherent shadowing of the brain hemisphere closer to the probe by the skull (Monteagudo and Timor-Tritsch, 2012; Cuingnet et al., 2013). On top of that, after exporting the images from the scanner to a Dicom format, we noticed that in some cases, the corpus callosum is facing to the left, and in others, to the right is the sagittal projection. Similarly, in some cases, structures such as the cavum septi pellucidi and the thalamus were facing to the left in the axial projection, while in some cases, they faced to the right. Thus, there were two non-identical brain orientations in our database. We believe that this is caused by different fetal positions in the womb during the ultrasound scan. In principle, one of these groups could have been mirrored to the orientation of the other. This is a feasible solution. However, we took a different approach and decided to use two different models that would fit the orientation of each group of subjects (for each age group). Selecting the first solution can reduce the chances of possible bias. Selecting the second would save processing steps. We choose the second approach. The reasons that led us to take the second approach were double. First, we wanted to reduce the pre-processing steps. Second, we wanted the two model brains for each age group to act as an additional test for our method's validity. If no bias between the results of the two models for the same age group is found, it suggests that our method is robust concerning the specific brain model used. Indeed, despite the risk of a bias in the ICV measurements due to the usage of two brain models for each age group, by comparing the results of ICV measurements based on these two models, we showed that the findings were not statistically different. Nevertheless, future researchers that will implement our pipeline can decide to take the first approach. Considering these two possible routes, we believe that this is not a severe limitation of our pipeline (or even an advantage).

A third limitation of our procedure is that the registration parameters had to be tweaked in some cases to obtain a satisfactory ICV measurement. As explained above, comparing the Minc-based and Elastix-based results provided a clear-cut criterion to decide whether the registration was successful. In cases that it is not, the operator can decide to tweak the parameters of either the Minc-based pipeline or the Elastix-based pipeline to achieve better measurements. In addition, a visual inspection of the ICV outline overlaying the scan, which is automatically produced, can inform the operator which of the two pipelines missed. In these cases, although identifying which registration parameters should be changed can be a bit tedious, it should be relatively easy for an experienced operator. It should be noted that one of the dangers of an automatic method that may include tweaking steps for the parameters is that it is downgraded from a fully automated level to a semi-automated process. The method described in this manuscript can undoubtedly suffer from such limitations. It necessitates some acquaintance with the pipelines and some learning phase. Once such a learning phase is fulfilled, choosing which parameter to tweak is relatively straightforward. We describe the main parameters that should be tweaked in the method section (see above). As a matter of fact, these were all the parameters we tweaked in this test cohort.

A fourth limitation of our method is related to the time it takes to run the pipeline (about half an hour per subject for the Minc-based pipeline and about 50 min for the Elastic-based pipeline). However, the method outweighs the time to complete a manual segmentation. Manual segmentation of one subject usually takes about 10 min. This duration is shorter than the time it takes the pipelines to run. However, it is a dead time from the point of view of the human rater. In the case of the automatic method presented in this manuscript, the most manually labor-intensive steps are those related to exporting the Dicom ultrasound scans to nifti files and sorting them into the two “Left” and “Right” groups. We have added some auxiliary python scripts that automatically take care of all other steps in preparing the dataset for analysis and reading the final results. Nevertheless, developing an automated method to export the ultrasound scans and deal with the two head orientations is left for future work.

## Data availability statement

The data that support the findings of this study are available on request from the corresponding author. The data are not publicly available due to privacy or ethical restrictions.

## Ethics statement

The studies involving human participants were reviewed and approved by Medical Research Ethics Committee Utrecht. Written informed consent to participate in this study was provided by the participants' legal guardian/next of kin.

## Author contributions

YC devised the experiment, developed the method, implemented the algorithm, analyzed the results, and wrote the manuscript. IJI carried out all the automatic-based experiments, analyzed the data, and wrote the manuscript. SMCdZ devised the experiment, helped in analyzing the data, and reviewed the manuscript. RL carried out all the manual experiments and reviewed the manuscript. MNB and RdH supervised the ultrasound measurements, provided expertise of fetal brain anatomy, and reviewed the manuscript. HHP devised the experiment and reviewed the manuscript. All authors contributed to the article and approved the submitted version.

## Funding

YOUth is funded through the Gravitation Program of the Dutch Ministry of Education, Culture, and Science and the Netherlands Organization for Scientific Research (NWO Grant No. 024.001.003).

## Conflict of interest

The authors declare that the research was conducted in the absence of any commercial or financial relationships that could be construed as a potential conflict of interest.

## Publisher's note

All claims expressed in this article are solely those of the authors and do not necessarily represent those of their affiliated organizations, or those of the publisher, the editors and the reviewers. Any product that may be evaluated in this article, or claim that may be made by its manufacturer, is not guaranteed or endorsed by the publisher.
