# Supplementary material for: Automatic measurements of fetal intracranial volume from 3D ultrasound scans
Source: Front Neuroimaging. 2022 Nov 4;1:996702. doi: 10.3389/fnimg.2022.996702 (PMC10406279; doi:10.3389/fnimg.2022.996702)
Supplement: Supplementary file 1 [file Data_Sheet_1.pdf]

Automatic Measurements of Fetal Intracranial Volume from 3D Ultrasound Scans - Supplementary Information

1 SUPPLEMENTARY METHODS

1.1 Manual Segmentation

Manual tracings were measured in the 4DView software version 18.2 of GE Healthcare using the VOCAL algorithm (https://voluson- sw.gehealthcare.com). For each ultrasound scan, all sweeps were displayed in a multiplanar view and rotated into the following orientation: transthalamic coronal view in up-left panel (A), midsagittal view in up-right panel (B), and axial midline transthalamic view in down-left panel (C). In panel B, the anterior brain was situated towards the left. In this orientation, the corpus callosum and cerebellar vermis should be visible in plane B and the hippocampus gyrus in plane C. Once all sweeps were correctly oriented, the sweep with the best quality was selected for further measurement. Intracranial volume was measured on plane C using VOCAL with 30° rotation steps. That is, the inner boundaries of the skull were manually traced every 30 degrees, over six planes. Afterward, the program automatically reconstructed the 3D representation of intracranial volume and calculated the volume in cm^3. Such a manual tracing procedure for calculating the ICV was previously shown to be highly reliable in terms of inter-rater and between-raters Intraclass Correlation Coefficients (see Albers et al. (2018)). An example of a manual tracing of the ICV is shown in Fig. S1 of the Supporting information.

1.2 Automatic Segmentation

1.2.1 Registration Algorithm Parameters

For the Minc-based pipeline, an additional pre-registration step was sometimes added. In these cases, the two scans were blurred before the affine registrations. Subsequently, all affine registration steps used the blurred images instead of the original ones. For the Elastix-based pipeline, the algorithm started with a translation-only registration step between the subject's scan and the brain model. Next, a rigid registration was applied between the subject's scan and the brain model (with the translation registration as an initial transformation). The rigid transformation included translation and rotation (Euler). As mentioned in the main text, in some cases, the rigid registration step was replaced by a similarity registration step that included also a global scaling. In addition, the Elastix-based algorithm controlled for the amount of blurring before each affine transformation separately for each step and not globally for all affine registration steps, as in the Minc-based pipeline case.

Concerning the B-spline registration steps, for the Minc-Based algorithm, the non-linear registrations were controlled using the -nonlinear, -iterations, -sub_lattice, -weight, -stiffness, and -similarity_cost_ratio flags to the minctracc application of the MINC-Toolkit (defaults - corrcoeff, {5,8,10}, 6, 1.0, 1.0, 0.3). Here, {} represents a parameter value to a different iterations of the registration. In addition, we used blurring before each non-linear registration step controlled by mincblur (default - {16,8,2}). For the Elastix-based non-linear registrations, we used as the key parameters the flags (defaults in parenthesis): Transform (BSplineTransform), Metric (AdvancedMattesMutualInformation), NumberOfHistogramBins (32), FixedLimitRangeRatio (0.01), MovingLimitRangeRatio (0.01),

FixedKernelBSplineOrder (3), MovingKernelBSplineOrder (3), NumberOfResolutions (5), ImagePyramidSchedule ({3 3 3 2 2 2 2 2 2 2 2 2 2 2 1 1 1}, {2 2 2 2 2 2 2 2 2 2 1 1 1 1 1 1}, {2 2 2 1 1 1 1 1 1 1 1 1 1 1 1}, {2 2 2 1 1 1 1 1 1 1 1 1 1 1 1}), MaximumNumberOfIterations (2000), ImageSampler (RandomCoordinate), NumberOfSpatialSamples (3000), FinalGridSpacingInPhysicalUnits ({3.0 3.0 3.0}, {2.0 2.0 2.0}, {2.0 2.0 2.0}, {1.0 1.0 1.0}), BSplineInterpolationOrder ({1}, {1}, {2}, {3}), FinalBSplineInterpolationOrder (3), SP_a (200.0), SP_A (50.0), SP_alpha (0.602).

In general, for the Minc-toolkit implementation, we have used three consecutive affine registrations and three consecutive B-spline registrations. For the Elastix-based pipeline, we used three consecutive affine registrations and four consecutive B-spline registrations. However, the Elastic-based pipeline allows choosing a smaller number of affine or B-spline registration steps.

REFERENCES

Albers, M. E. W. A., Buisman, E. T. I. A., Kahn, R. S., Franx, A., Onland-Moret, N. C., and de Heus, R. (2018). Intra- and interobserver agreement for fetal cerebral measurements in 3D-ultrasonography. *Human Brain Mapping* 39, 3277–3284. doi:10.1002/hbm.24076

2 SUPPLEMENTARY TABLES

Cross-sectional data					
Scan period	# of subjects (Females)	# Subjects Model Group Left/Right	Gestational age (range) (days)	# of subjects passed QC	# Subjects Model Group Left/Right that passed QC
20 Weeks	92 (42)	42 (18)/50 (24)	152.9 (140-170)	92 (42)	42 (18)/50 (24)
30 Weeks	90 (39)	42 (15)/48 (24)	213.6 (203-230)	86 (39)	39 (15)/47 (24)
**Combined time points data**					
	# of subjects (Females)	# of subjects passed automatic QC	-	-	-
Total Subjects	98 (43)	94 (43)	-	-	-
Two repeated measurements	84 (38)	80 (38)	-	-	-

Table S1. Statistical Characteristics of the validation cohort. Fatal brains were imaged using a Voluson E10 machine at two different time points. First, fetuses were imaged around GA of 20 weeks. Second, most of the fetuses' brains and some additional ones were imaged around GA of 30 weeks. Upper part of the table - statistical characteristics at the two different time points. Lower part of the table - combined time points data. Parenthesis - the number of females. QC - Quality Control.

20 weeks							
	Intercept			Slope			R^2
	Value±SD	t-value	P-Value	Value±SD	t-value	P-Value	
Left Group	17±5	3.4	0.001	0.82±0.6	13.8	<2e-16	0.82
Right Group	-1.2±3.8	-0.3	0.756	0.98±0.04	21.8	<2e-16	0.91
All subjects	8.0±3.6	2.3	0.022	0.9±0.04	21.6	<2e-16	0.84
30 weeks							
	Intercept			Slope			R^2
	Value±SD	t-value	P-Value	Value±SD	t-value	P-Value	
Left Group	1.4±18	0.075	0.940	0.99±0.07	15.0	<2e-16	0.85
Right Group	4.3±10	-0.44	0.666	0.98±0.03	27.2	<2e-16	0.94
All subjects	3±10	0.35	0.727	0.98±0.03	28.4	<2e-16	0.90

Table S2. Elastix and Minc Correlation. Parameters of the linear fits between the results of the Elastix-based pipeline and the Minc-based pipeline. Upper table is for the GA of 20 weeks (corresponding to Fig. 4(a) of the main text). Lower table is for the GA of 30 weeks (corresponding to Fig. 5(a) of the main text).

20 weeks							
	Intercept			Slope			R^2
	Value±SD	t-value	P-Value	Value±SD	t-value	P-Value	
Left Group	8.5±3.8	2.2	0.03	0.98±0.05	20.4	<2e-16	0.91
Right Group	5.8±3.8	1.5	0.133	1.0±0.05	20.5	<2e-16	0.90
All subjects	7.3±2.7	2.7	0.008	1.0±0.03	29.0	<2e-16	0.90
30 weeks							
	Intercept			Slope			R^2
	Value±SD	t-value	P-Value	Value±SD	t-value	P-Value	
Left Group	40±26	1.5	0.134	1.0±0.10	9.1	5e-11	0.68
Right Group	-17±19	-0.89	0.377	1.2±0.08	15.4	<2e-16	0.84
All subjects	10±16	0.67	0.507	1.1±0.07	16.4	<2e-16	0.76

Table S3. Automatic and Manual Measurements Correlation. Parameters of the linear fits between the results of the automatic measurements of the ICV and the manual traced ICV for the same subjects. Upper table is for the GA of 20 weeks (corresponding to Fig. 4(b) of the main text). Lower table is for the GA of 30 weeks (corresponding to Fig. 5(b) of the main text).

3 SUPPLEMENTARY FIGURES

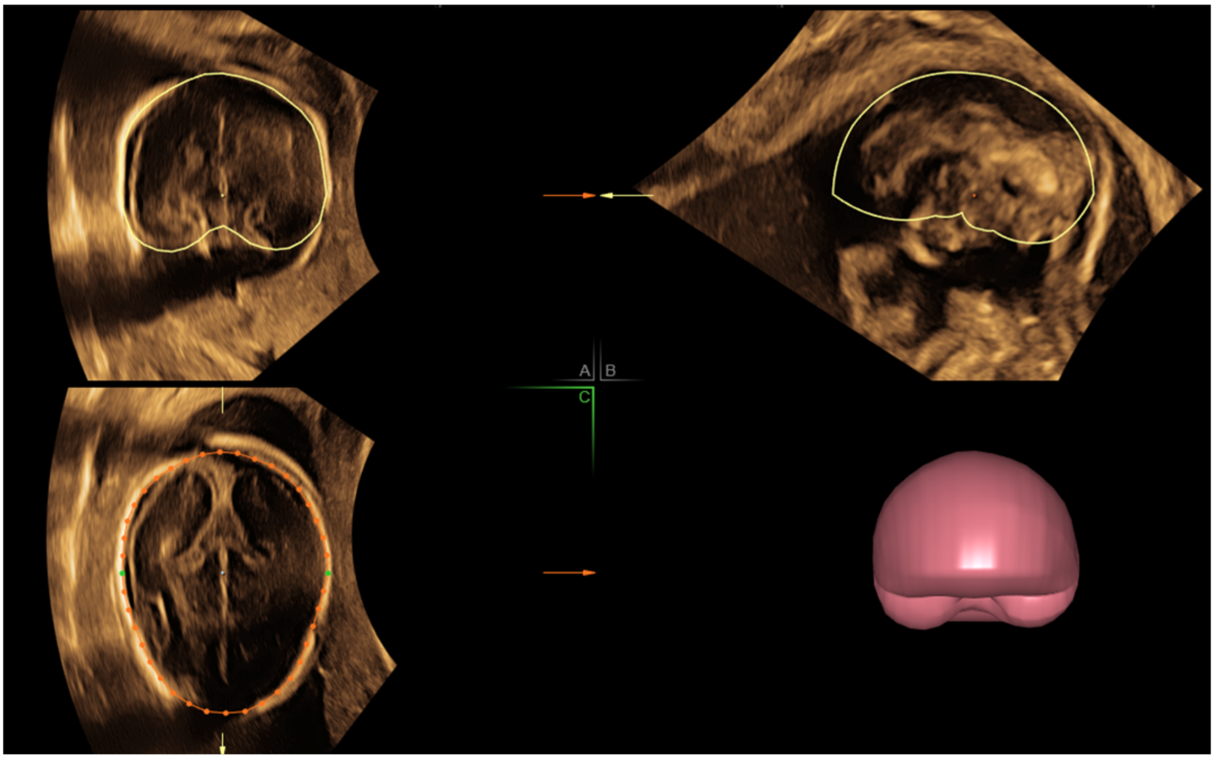

Figure S1. Manual tracing of the ICV. An example of the manual tracing of the ICV using the VOCAL method for one of the subjects. Tracing started in the axial plane (plane C in the figure) and advanced through steps of 30°. The end result of the ICV as a volume structure appears in the lower right panel of the figure. The tracing was done using the VOCAL plugin of the ultrasound 4DView program.

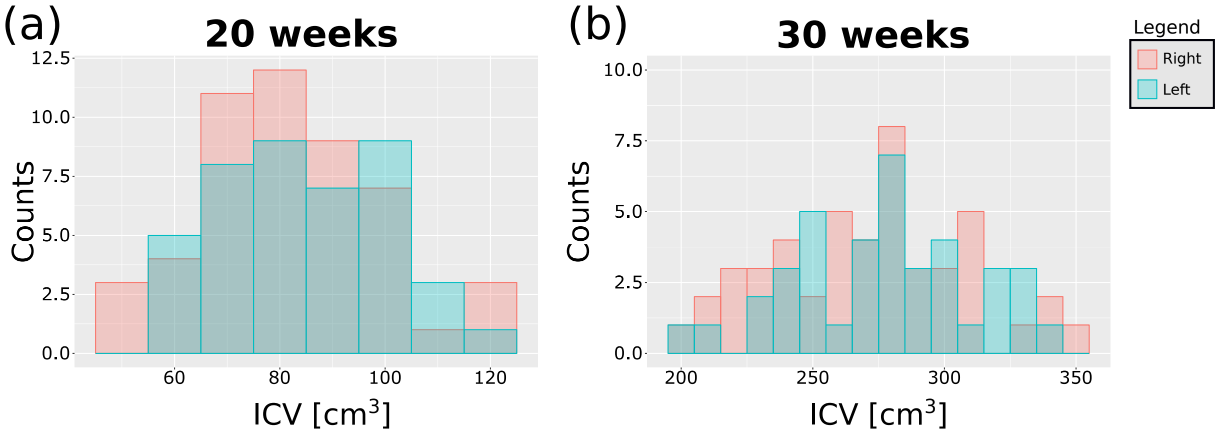

	20 Weeks		30 Weeks	
	Value±SD	2-sided test P-value	Value±SD	2-sided test P-value
Left Group	85±16	0.466	277±34	0.477
Right Group	83±16		271±37	

Figure S2. Statistical comparison of the two brain models. Distributions of the automatically calculated ICV results for the two brain models ('Left' in pink and 'Right' in sky blue) for GA of 20 weeks (a); and GA of 30 weeks (b). The table below the graphs provides the average of each brain model, SD of each brain model, and P-value of a t-test comparison between the two brain models for each age group.

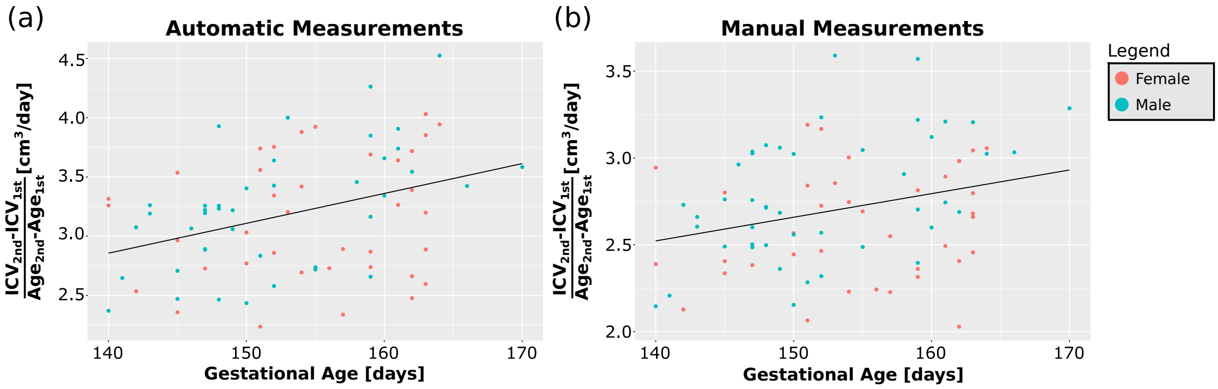

	Intercept			Slope			
	Value±SD	t-value	P-value	Value±SD	t-value	P-value	R^2
Automatic	-0.7±1.1	-0.585	0.560	0.03±0.007	3.37	0.001	0.117
Manual	0.6±0.8	0.791	0.432	0.01±0.005	2.67	0.009	0.070

Figure S3. Growth Rate Plot. Plots of the rate of ICV growth for each individual as a function of the GA at the first measurement for the automatically calculated ICV (a) and the manually traced ones (b). Solid lines - linear fit of the results for males and females together. The tables below the graphs provide the fitting parameters of the linear fits.
